# Robot-assisted lung segmentectomy in a patient with an implantable left ventricular assist device: a case report

**DOI:** 10.1186/s44215-025-00213-6

**Published:** 2025-07-09

**Authors:** Motoka Omata, Shota Mitsuboshi, Hiroaki Shidei, Akira Ogihara, Hiroe Aoshima, Tamami Isaka, Hidetoshi Hattori, Shinichi Nunoda, Junichi Yamaguchi, Hiroshi Niinami, Masato Kanzaki

**Affiliations:** 1 Department of Thoracic Surgery, Tokyo Women’s Medical University, 8-1 Kawada-Cho, Shinjuku-Ku, Tokyo , 162-8666 Japan; 2 Department of Cardiology, Tokyo Women’s Medical University, 8-1 Kawada-Cho, Shinjuku-Ku, Tokyo, 162-8666 Japan; 3 Department of Cardiovascular Surgery, Tokyo Women’s Medical University, 8-1 Kawada-Cho, Shinjuku-Ku, Tokyo, 162-8666 Japan

**Keywords:** Left ventricular assist device; LVAD, Bridge to transplant; BTT, Lung cancer, Sublobar resection

## Abstract

**Background:**

Survival can be prolonged in patients with end-stage heart failure using left ventricular assist devices (LVADs); however, increased longevity raises the risk of developing noncardiac complications, including malignancies requiring surgery.

**Case presentation:**

A 58-year-old man with an LVAD was referred for the diagnosis and treatment of an undiagnosed nodule in the right upper lobe, which was detected during a preoperative computed tomography (CT) scan, for heart transplantation assessment. CT revealed a 9-mm nodule in the right anterior segment (S^3^), and an 18F-fluorodeoxyglucose positron emission tomography showed significant uptake, suggestive of lung cancer. A robot-assisted thoracoscopic right S^3^ segmentectomy was performed. Intraoperative hemodynamic monitoring included an arterial line, a central venous catheter, a pulmonary arterial catheter, and transesophageal echocardiography. The procedure was completed successfully without complications. Pathological analysis confirmed adenocarcinoma, classified as pathological stage T1aN0M0 (Stage IA1). The patient subsequently underwent heart transplantation and LVAD removal on postoperative day 185.

**Conclusions:**

A patient with lung cancer and an LVAD who was awaiting heart transplantation successfully underwent robot-assisted thoracoscopic right S^3^ segmentectomy, enabling him to subsequently undergo a heart transplant.

## Background

Left ventricular assist devices (LVADs) have become essential for managing end-stage heart failure, particularly in patients awaiting heart transplantation. Technological advancements have reduced complication rates, enabling patients to survive longer with mechanical circulatory support. However, extended survival is associated with an increased risk of developing noncardiac comorbidities, including malignancies that may necessitate surgical intervention. We present the case of a patient with an LVAD who successfully underwent robot-assisted thoracoscopic right anterior segment (S^3^) segmentectomy for lung cancer, followed by heart transplantation.

## Case presentation

A 58-year-old man with an EVAHEARTII® LVAD (Sun Medical Technology Research Corp., Nagano, Japan) was referred for the diagnosis and treatment of a small, undiagnosed nodule in the right upper lobe. He had a history of severe ischemic cardiomyopathy and underwent cardiac resynchronization therapy (CRT) with a CRT-defibrillator (CRT-D) device 7 years earlier. Because of progressive cardiac deterioration, he received the EVAHEARTII® LVAD 5 years ago as a bridge to heart transplantation.

**Fig. 1:**
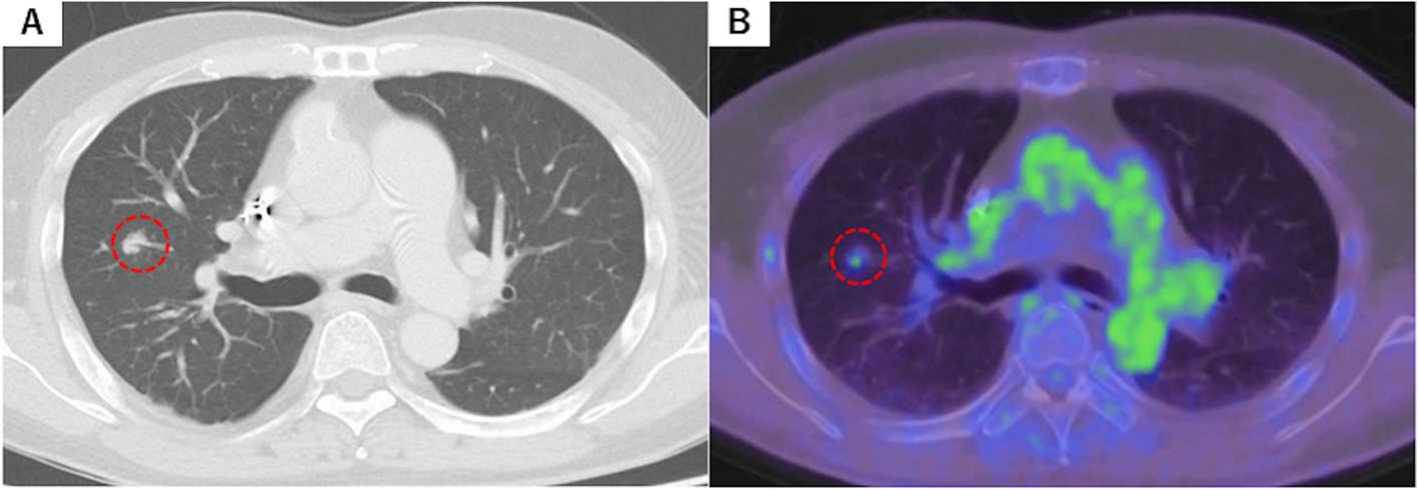
Computed tomography (CT) and 18F-fluorodeoxyglucose positron emission tomography (FDG-PET). **A** CT scan showing a 9-mm nodule in the right anterior segment. **B** FDG-PET scan showing substantial accumulation of isotopes in the tumor (SUVmax = 1.39)

**Fig. 2:**
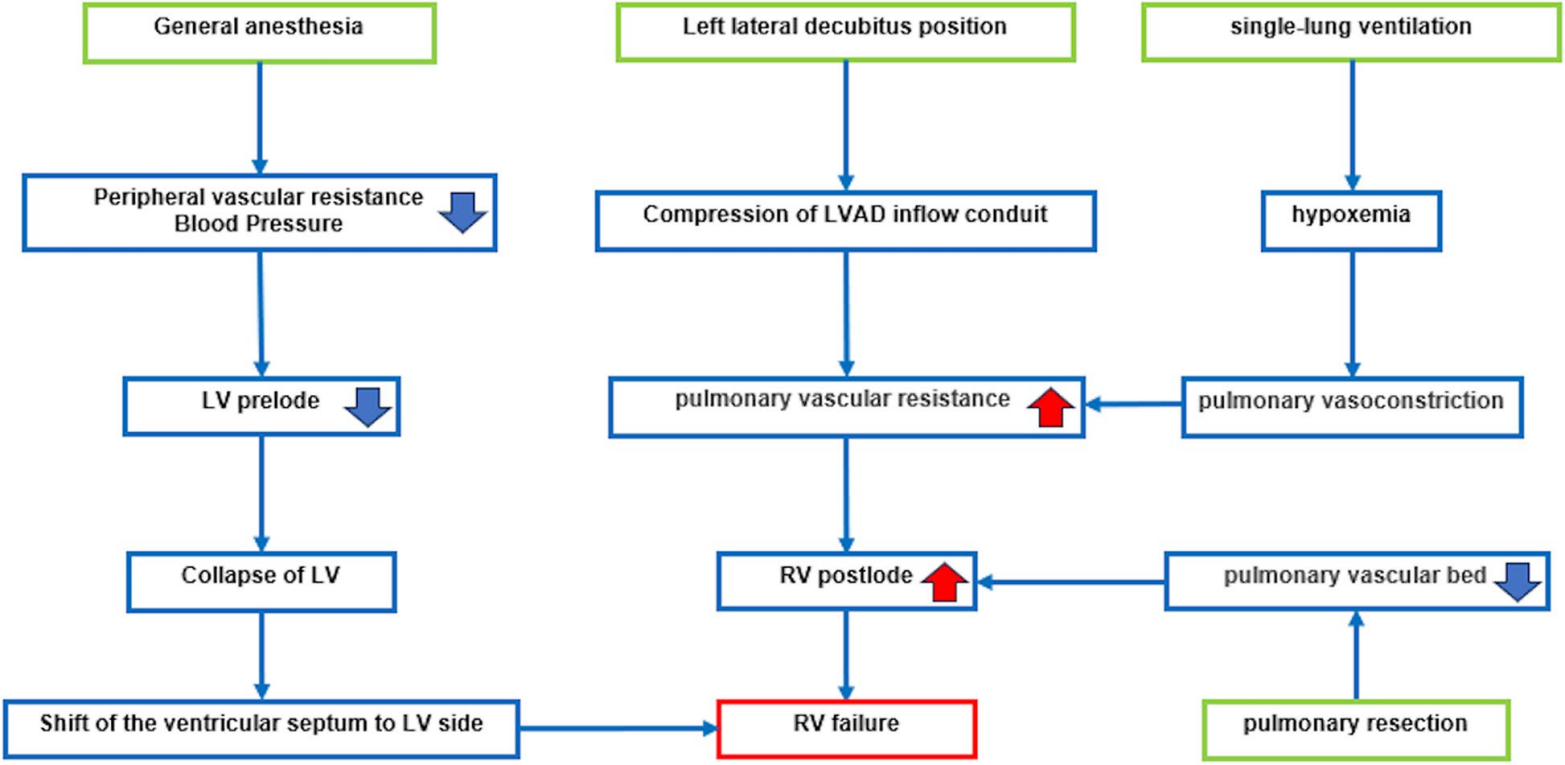
Right ventricular failure risk in patients with left ventricular assist devices (LVAD) undergoing lung surgery. Lung surgery in a patient with LVAD poses various risks, including left ventricular collapse from reduced preload caused by general anesthesia, septal shift toward the LV, and decreased LVAD flow from compression of the inflow conduit in the lateral decubitus position—particularly on the left side. Additional risks include right heart failure from increased pulmonary vascular resistance and right ventricular deformation, hypoxia from single-lung ventilation, pulmonary vasoconstriction, and increased right heart load due to reduced pulmonary vascular bed following lung resection [1–4]. LVAD, left ventricular assist device; LV, left ventricle; RV, right ventricle

As part of the preoperative evaluation for heart transplantation, a computed tomography (CT) scan revealed a 9-mm nodule in the right S^3^ (Fig. 1). An 18F-fluorodeoxyglucose positron emission tomography (FDG-PET) scan showed significant isotope uptake in the lesion, with a maximum standardized uptake value (SUVmax) of 1.39 (Fig. 2). To assess eligibility for heart transplantation and establish a definitive diagnosis and treatment plan for the lung nodule, the patient was referred to our thoracic surgery department.

Transthoracic echocardiography and right heart catheterization showed no signs of pulmonary hypertension or significant right heart dysfunction. Given the suspicion of malignancy, a robot-assisted thoracoscopic right S^3^ segmentectomy was planned. Preoperatively, clopidogrel sulfate and warfarin were discontinued, and anticoagulation was managed with a heparin infusion. Aspirin continued throughout the perioperative period.

During the operation, hemodynamic monitoring was conducted using an arterial line, central venous catheter, pulmonary arterial catheter, and transesophageal echocardiography. The EVAHEARTII® pump speed was maintained at 1,840 to 1,850 rpm, with a power consumption of 3.0–3.4 W. The CRT-D device was programmed to the standard DDD pacing mode at a rate of 90 beats per min (bpm.). A double-lumen endotracheal tube was inserted, and the patient was positioned in the left lateral decubitus position. Intraoperative hemodynamics showed an arterial pressure of approximately 80/60 mmHg, central venous pressure around 15 mmHg, and mean pulmonary artery pressure around 20 mmHg.

The robot-assisted thoracoscopic right S^3^ segmentectomy was completed successfully. The total operative time was 170 min, with an estimated blood loss of 26 mL. The endotracheal tube was removed 4 h after the patient was admitted to the intensive care unit. Heparin infusion was resumed 5 h after rgery, after confirming the absence of intrathoracic bleeding. The chest tube was removed on postoperative day 4, and warfarin therapy was restarted. The patient had an uneventful recovery and was discharged on postoperative day 24.

Pathological evaluation confirmed adenocarcinoma with an invasive diameter of 10 mm and no lymph node metastasis, and it was classified as pathological stage T1aN0M0 (Stage IA1). Subsequently, the patient underwent heart transplantation and LVAD removal on postoperative day 185, followed by CRT-D extraction on postoperative day 206.

## Discussion and conclusions

Ventricular assist devices (VADs) are designed to replace the pumping function of a failing heart and maintain systemic circulation in patients with heart failure. The devices serve multiple roles, including a bridge to transplantation (BTT), a bridge to candidacy, a bridge from extracorporeal VAD to implantable VAD (bridge to bridge), a bridge to recovery, and destination therapy [5].

In Japan, the average waiting time for heart transplantation has been steadily increasing, currently at 1877 days for adults. Since the approval of the implantable continuous-flow ventricular assist device (iVAD) for BTT in 2011, the number of iVAD-based BTT cases has increased significantly. Of the 636 patients awaiting heart transplantation, 611 have undergone iVAD implantation, highlighting the pivotal role of iVAD in BTT [6].

During the heart transplantation waiting period or preoperative screening, malignancies may be discovered, potentially affecting transplant eligibility. Studies have reported that 2%–3% of patients on the heart transplant waiting list develop malignancies, with the most common being gastrointestinal, lung, and skin cancers. The presence of a malignant tumor is typically considered a contraindication for heart transplantation because of the risk of cancer recurrence or secondary malignancy resulting from immunosuppressive therapy [7, 8].

For patients requiring heart transplantation, accurate pathological diagnosis and tumor staging are crucial. This ensures that the tumor recurrence risk is carefully evaluated before the start of transplantation [1]. When performing pulmonary resection in patients with an LVAD, several factors must be considered, as given below [1–4].

### Operative care

Lung surgery is typically performed in the lateral decubitus position with one-lung ventilation. This position, however, may lead to inadequate preload to the LVAD [1, 9]. Variations in the robot-assisted thoracoscopic surgery (RATS) procedure include differences in the number of robotic arms, the use of carbon dioxide insufflation, additional utility incisions, and port locations. For lung malignancies, lung resection using four robotic arms is common. The assistant port is usually positioned on the lower limb side of the robotic arms in the narrow space between the robotic arms and the pelvis. In LVAD patients, careful consideration is needed for the LVAD driveline, which passes through tight spaces. Bed-side surgeons must be mindful of its presence when assisting with surgery or exchanging robotic forceps. To minimize disruption and reduce driveline manipulation, we placed the assistant port in the 4th or 5th intercostal space anteriorly, cranial to the robotic arm. This positioning helps reduce the frequency of robotic forceps exchanges during RATS and minimizes potential complications associated with the driveline.

During surgery, the EVAHEARTII® pump speed was maintained at 1,840 to 1,850 rpm, and the heart rate was fixed at 90 bpm by the CRT-D. It is essential to avoid LVAD dysfunction caused by driveline occlusion and to prevent contamination of the LVAD driveline during the procedure.

### Careful management of anticoagulants

Patients with LVAD typically require anticoagulants (e.g., warfarin and direct oral anticoagulants) and antiplatelet therapy (e.g., aspirin) to prevent thromboembolism. Preoperatively, anticoagulants should be replaced with heparin, which is discontinued immediately before surgery. Postoperatively, the anticoagulant prescription must be promptly resumed after confirming that there is no bleeding risk.

In this case, clopidogrel sulfate and warfarin were discontinued 7 days before RATS, and the patient was switched to heparin infusion, which was discontinued immediately before RATS. No active bleeding was detected within the thoracic cavity, and heparin infusion was resumed 5 h after RATS. Aspirin was administered continuously during the perioperative period.

### Adequate hemodynamic management

Monitoring LVAD function and assessing right heart performance—particularly, tricuspid regurgitation—using transesophageal echocardiography is critical. Central venous pressure, pulmonary artery pressure, and systemic blood pressure should be carefully managed to maintain adequate LVAD flow. In this case, the pressures during surgery were as follows: arterial pressure of 80/60 mmHg, a central venous pressure of 15 mmHg, and mean pulmonary artery pressure of 20 mmHg. These values were comparable to those obtained at the time of the preoperative examination. LVAD blood flow was stable during surgery, and the RATS right S^3^ segmentectomy was performed without any LVAD-related complications. From a hemodynamic perspective, minimally invasive procedures and sublobar resections are preferable to reduce risk.

### Multidisciplinary approach

Given the high risk of pulmonary resection in patients with LVAD, cooperation across multiple departments and disciplines is essential. Thoracic surgeons, anesthesiologists, cardiologists, cardiac surgeons, intensive care unit staff, nurses, and clinical engineers must work together to ensure optimal care and outcomes.

We selected RATS right S^3^ segmentectomy because the robotic surgical system reduced the risk of intraoperative bleeding by providing a clear view of the surgical field and enabling precise manipulation with robotic forceps. Additionally, the tumor was small (9 mm), and the increase in right ventricular afterload due to the reduction in the pulmonary vascular bed after resection was minimized. This approach resulted in minimal blood loss, and the patient had an uneventful postoperative recovery. Throughout the surgery, LVAD flow was consistently maintained, with optimal hemodynamic management under careful circulatory monitoring. This case demonstrated that pulmonary resection played a crucial role not only in the successful treatment of lung cancer but also in supporting the patient through to heart transplantation, underscoring the importance of a multidisciplinary approach in complex cases.

## Data Availability

All the data and materials supporting our findings are included within the article.

## Abbreviations

LVAD: Left ventricular assist device
S^3^: Anterior segment
CRT: Cardiac resynchronization therapy
CRT-D: Cardiac resynchronization therapy defibrillator
CT: Computed tomographic
FDG-PET: 18F-fluorodeoxyglucose positron emission tomography
SUVmax: Maximum standardized uptake value
VAD: Ventricular assist devices
BTT: Bridge to transplantation
iVAD: Implantable continuous-flow ventricular assist device
RATS: Robot-assisted thoracoscopic surgery
